# On computation of information entropy measures for certain fractal molecular architectures

**DOI:** 10.3389/fchem.2025.1677841

**Published:** 2025-10-15

**Authors:** S. Roy, P. Noah Antony Daniel Renai, K. Jyothish

**Affiliations:** 1 Department of Mathematics, School of Advanced Sciences, Vellore Institute of Technology, Vellore, India; 2 Department of Science and Humanities, KGiSL Institute of Technology, Coimbatore, India

**Keywords:** degree-based entropies, topological indices, kekulene, terpyridine complex, fractal molecular structures

## Abstract

Fractal molecular structures constitute a distinct category of systems characterized by self-similarity and hierarchical growth patterns, which can be effectively examined by graph-theoretical methods. This manuscript introduces a refined adaptation of Shannon’s entropy calculation method by evaluating the degree-based topological indices for fractal molecular structures, specifically Kekulene $\left(KE_{n}\right)$ and Terpyridine Complex Sierpinski Triangle $\left(SE_{n}\right)$ systems, and examines their physicochemical implications. Builds upon previously reported hydrogen-bonded fractal architectures, we have generated graphical representations of these fractal molecular structures in the form of a Kekulene ring and a Sierpinski triangular system. Subsequent computational analysis enabled the determination of entropy values for degree-based topological descriptors. These calculations provide valuable insights into the structural complexity and molecular properties of the fractal systems, offering potential applications in quantitative structure-property relationship and quantitative structure-activity relationship analyses for advanced material design and synthesis.

## 1 Introduction

In chemical graph theory, a molecular graph is a representation of a molecule, where the edges signify chemical relationships between atoms and the vertices stand for atoms. Through the transformation of molecular structures into mathematical objects, this method makes the application of graph-theoretical concepts and methods easier. Topological indices are numerical values that represent different structural characteristics of the molecule and are derived from the molecular graph. Indices such as the Zagreb, Wiener, and Randić index provide information on reactivity, molecular stability, and other aspects of chemistry. Topological indices play a crucial role in cheminformatics by providing information on molecular size, branching, and connectivity. This information is useful in predicting biological activity, chemical behavior, and the design of novel compounds. In chemistry, fractal structures are useful because they facilitate the description and examination of intricate patterns and behaviors at different scales. Fractals have a wide range of applications, one of which is surface characterisation. Here, they are utilized to simulate surface imperfections and roughness at the atomic and molecular levels, which is essential for comprehending catalyst properties and reactions. Fractal analysis is useful in characterizing pore structures and their effects on adsorption, diffusion, and reactivity in porous materials, such as silica gels and zeolites. Additionally, fractals are essential to understanding reaction kinetics because they shed light on the uneven spatial distribution of reactive sites in heterogeneous catalysts, which affects reaction speeds and processes. Fractal principles are used in polymer chemistry to explain the features of fractal-like aggregates, network development, and the scaling behavior of polymer chains. Additionally, fractal models are employed to study diffusion processes in complex media, offering a more accurate depiction of how substances move through irregular structures. Overall, fractal structures offer a robust framework for exploring the complexities of various chemical systems (Weib et al., 2019). Computational studies on fractal chemical structures involve using computer-based methods to understand their structure, properties, interactions, and biological activities. Here are some possible computational studies and analysis on fractal based molecular structures that could be carried out to utilize the numerical invariants:

- Molecular Docking: Computational docking studies are commonly used to understand how fractal chemical architectures interact with biological targets such as enzymes, receptors, or proteins. By simulating the binding of fractal molecular structures to these targets, researchers can predict their potential biological activities and mechanisms of action.
- Quantum Chemical Calculations: Quantum chemical methods like density functional theory (DFT) or semi-empirical calculations are used to study the electronic structure, energy levels, and reactivity of fractal chemical architectures. These calculations provide insights into their stability, reactivity towards free radicals, and other chemical properties.
- Molecular Dynamics Simulations: Molecular dynamics (MD) simulations are used to study the dynamic behavior of fractal chemical architectures and their interactions with other chemical systems over time. These simulations can reveal information about the flexibility, conformational changes, and stability of fractal chemical complexes.
- Pharmacophore Modeling: Pharmacophore modeling involves identifying the essential structural features (pharmacophores) of certain fractal chemical architectures that are responsible for their reactivity. Computational methods can help in constructing pharmacophore models based on known bioactive fractal molecular structures and using them to design new compounds with desired properties.
- Virtual Screening: Virtual screening is a computational technique used to screen large libraries of compounds, including some chemical architectures, against specific biological targets. By using molecular docking or other methods, researchers can identify potential fractal chemical compounds with high binding affinity and selectivity for the target of interest.
- Network Pharmacology: Computational approaches such as network pharmacology can be applied to study the complex interactions between fractal structures, biological targets, and biological pathways. These studies can help in understanding the holistic effects of fractal molecular structures on biological systems and identifying synergistic interactions with other compounds.
- Quantitative Structure-Activity Relationship Studies: QSAR studies involve correlating the chemical structure of fractal chemical compounds with their biological activities using statistical and computational methods. These studies are useful for predicting the bioactivity of novel chemical derivatives and optimizing their structures for enhanced potency.

Studies on fractal supramolecular systems reveal that coordination-driven self-assembly is a key approach for designing supramolecular architectures, facilitating the evolution from simple two-dimensional macrocycles to intricate three-dimensional cage-like frameworks (Chichak et al., 2004; Hasenknopf et al., 1996; Olenyuk et al., 1999). Supramolecular chemistry, often characterized as “chemistry beyond the molecule,” is principally concerned with molecular recognition processes and the generation of higher-order assemblies mediated by non-covalent interactions. The reliance on such interactions allows supramolecular systems to function as modular entities, wherein discrete molecular units associate through well-defined binding motifs (Huang and Anslyn, 2015; Renai et al., 2025). These interactions are inherently multifaceted, encompassing both attractive and repulsive forces, most notably hydrogen bonding and metal–ligand coordination, which collectively underpin the stability and diversity of supramolecular assemblies (Roy et al., 2014).

According to a recent study the induced heat conduction within the polymers due to designing intermolecular interactions has been shown to be effective (Kim et al., 2015; Mehra et al., 2018a; Mehra et al., 2018b). Fine-tuning various non-covalent interactions in supramolecular structures could be another method for generating the best dissipating materials. In the development of supramolecular analysis, many hydrogen bonds are typically identified, which can operate as thermal highways for phonon transport. The capacity of supramolecular assembles to generate stable crystal structures is another important feature that qualifies them for thermal conduction. The thermal conduction properties of polymeric materials are strongly dependent on the morphology and dimensions of their crystalline domains (Mu et al., 2016). In supramolecular systems, the assembly of stable aggregates is frequently governed by non-covalent interactions involving complementary molecular motifs (Seto and Whitesides, 1990). Among various architectures, terpyridine-metal complex nanosheets in combination with Sierpinski triangle-type systems represent a particularly well-studied and versatile class of supramolecular assemblies.

The fractal geometric structures of terpyridine and kekulene molecules, such as the terpyridine complex Sierpinski triangle fractal and the kekulene fractal, have been examined in this study. Fractal structures are essentially complex geometric forms that exhibit self-similarity, or patterns that repeat at different scales. These forms are recognized for their intricate, frequently indefinitely detailed patterns that can be found in both mathematical constructions and natural environments. Fractals differ from conventional geometric shapes having whole number dimensions in that they have fractional or non-integer dimensions. Because of this property, fractals can effectively simulate and depict the asymmetrical, fragmented forms that can be found in the natural world, such as mountain ranges, coastlines, cloud formations, and tree branches. Iterative procedures are used to create fractals, where simple rules are repeatedly applied to create the overall complex structure. This iterative nature results in infinite detail, with each smaller part mirroring the larger structure. Fractals are useful in many branches of science and the arts because of their recursive and self-similar characteristics. They are employed in computer graphics to produce lifelike textures and sceneries. They aid in the understanding of complicated, dynamic processes in the study of chaotic systems. Therefore, fractal structures offer deep insights into the patterns and complexities of the world around us and constitute an intriguing intersection of mathematics, nature, and art. In order to improve our comprehension of chemical systems, mathematical chemistry is an interdisciplinary field that integrates chemical study and mathematical ideas and methods. This area of study models molecular structures, investigates reaction mechanisms, and predicts the features of novel compounds using a range of mathematical techniques, such as graph theory, linear algebra, and computational algorithms. Topological indices, commonly referred to as molecular descriptors, provide evidence linking important physicochemical and biological processes to molecular structure. These descriptors are a component of a theoretical instrument that characterizes the molecular structures. Topological indices represent the underlying connections in molecular networks as structure-based invariants associated with molecular graphs. In QSAR and QSPR investigations, topological indices are frequently employed as an effective tool. Their application in these areas has led to increased attention and recognition in recent years. In this work we have computed the general mathematical expression using various degree-based topological indices for the kekulene fractal and terpyridine complex Sierpinski triangle fractal. Also, along with that we have calculated the information entropy, namely Shannon entropy for these two fractal geometry structures (Ma et al., 2006; Ma et al., 2007; Ida et al., 2008; Yan et al., 2009; Gao and Yan, 2018; Bauer et al., 2011; Payamyar et al., 2014).

## 2 Materials and methods

### 2.1 Sierpinski triangle system

**FIGURE 1:**
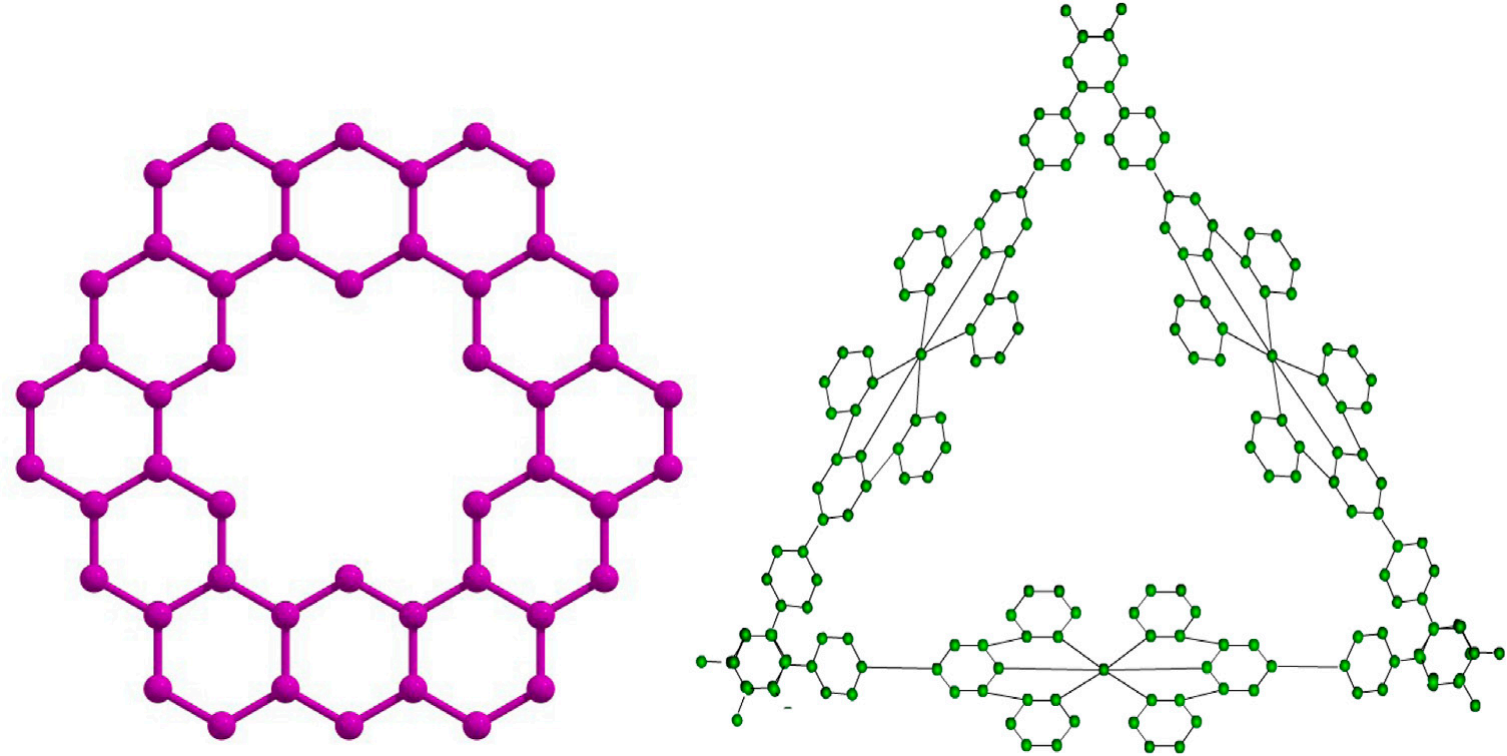
Kekulene and Terpyridine complex Sierpinski triangle fractal first growth stage.

Honoring Polish mathematician Waclaw Sierpinski, the Sierpinski triangle, also referred to as the Sierpinski gasket is a fractal structure. An equilateral triangle is created by recursively splitting it into smaller equilateral triangles, taking off the center triangle at each stage. This results in the formation of a self-similar pattern that is structurally stable at all magnification levels. The Sierpinski triangle fractal illustrates infinite complexity in a finite space and serves as an excellent example of recursion theory. Its uses in a variety of domains, including as computer graphics, antenna design, and the investigation of natural phenomena like snowflakes and crystal formation, demonstrate the fusion of mathematics and environment. Each fractal molecule can be regarded as a crystalline complex consisting of a finite set of atoms (vertices) and hydrogen bonds (edges), arising from the coordination of terpyridine (*TPY*) ligands with metal centers such as *Fe* or *Co* in a threefold arrangement. From a graph-theoretical perspective, atoms correspond to vertices while hydrogen bonds are represented as edges. The resulting fractal molecular complexes incorporate regular cyclic substructures of length six $\left(C_{6}\right)$ and five $\left(C_{5}\right)$ . The initial dimensional form of such a fractal complex is depicted in Figure 1. More generally, the n -dimensional fractal molecular complex is denoted as the terpyridine-based Sierpinski fractal $SE_{n}$ (Weib et al., 2019), as illustrated in Figure 3.

### 2.2 Kekulene ring

Kekulene is an intriguing polycyclic aromatic hydrocarbon that is distinguished by its distinct and extremely symmetrical structure, which is made up of twelve benzene rings united in a circular pattern. Accordingly, it is categorized as a circulene having the molecular formula $C_{48}H_{24}$ . It was first synthesized in 1978 and given the name after August Kekulé in honor of the German chemist who is renowned for proposing the ring structure of benzene. With its symmetry and cyclic planar structure, kekulene serves as just a larger form of benzene. The molecule is a prime example of aromaticity, a property in which the stability of a molecule is increased by electron delocalization within it. Ever since Kekulé presented the cyclic structure of benzene in 1865, chemists have been fascinated by this fundamental idea in organic chemistry. Kekulene is a classic example of a cycloarene, has two concentric macrocyclic pathways called annulene and annulene. One stabilization mechanism for Kekulene involves π -conjugation. This phenomenon has been called superaromaticity, and it has generated plenty of discussion throughout the years. In accordance with Clar’s rule, which specifies that in benzenoid compounds, the number of distinct aromatic benzene rings should be maximized and the number of double bonds should be minimized, an alternate model proposes that kekulene is composed of six distinct aromatic sextets. Due to its conjugated π -electron system, they exhibit intriguing electronic features, including counter-loop currents and physical characteristics, that are of interest in theoretical chemistry and materials science.

### 2.3 Terpyridine system

G. Morgan and F. H. Burstall originally synthesized terpyridine, a heterocyclic molecule generated from pyridine, in 1932 by oxidatively combining pyridines. Terpyridine (2,2′; 6′,2″-terpyridine, often abbreviated as Terpy or Tpy) is referred to be a tridentate ligand because it binds at three meridional locations to produce two neighboring 5-membered $MN_{2}C_{2}$ chelate rings in stable complexes with a range of metal ions. In accordance with other polypyridine compounds such as 1,10-phenanthroline and 2,2′-bipyridine, terpyridine forms complexes with the majority of transition metal ions. These complexes are recognized for their unique electrochemical and optical characteristics, including substantial absorbance, reversible reduction and oxidation, and metal-to-ligand charge transfer in the visible spectrum. Three nitrogen donor atoms link to the metal center in a triangle arrangement to generate meridional complexes, which are facilitated by the stiff, planar structure of terpyridine. This unique coordination geometry often imparts exceptional properties to the complexes, including intense luminescence, efficient electron transfer, and catalytic activity. Consequently, terpyridine is a crucial component in the creation of supramolecular architectures, functional materials, and catalysts, with potential applications in sensing, solar energy conversion, and medicine. Additionally, terpyridines are widely used to complex transition metal ions and serve as building blocks for supramolecular gel systems (Dlubak et al., 2012; Noah and Roy, 2022; Novoselov et al., 2004; Sakamoto et al., 2015; Zhao et al., 2020).

### 2.4 Degree-based topological indices

In the context of QSPR analysis, the application of complex chemical compounds has been accompanied by a notable growth in the development and utilization of topological indices, which serve as effective tools for correlating and classifying the physicochemical properties of molecules. Among their various applications, QSPR and QSAR studies are the most prominent (Gute et al., 1999; Hayat et al., 2019; Renai and Roy, 2023; Viswanadhan et al., 2001). Topological indices, being structure-based invariants of molecular graphs, capture the intrinsic connectivity patterns of molecular networks and, as such, have attracted considerable attention in recent years for their utility in QSPR models (Balasubramanian, 2022; Balasubramanian et el., 2018; Balasubramanian, 2021; Jyothish and Roy, 2024; Jyothish and Roy, 2025; Kalaam and Greeni, 2025). When combined with entropy-based descriptors, these indices provide a powerful framework for QSPR and QSAR analyses. Notably, in several classes of natural chemical systems, entropy-related quantities exhibit direct correlations with fullerene properties (Gutman and Tosovic, 2013). Based by these insights, we extend our study to the computation of entropy measures for fractal molecular complexes to explore their potential applications.

To establish a mathematical framework, we introduce certain notations that will be employed throughout the paper. Let G denote the molecular graph corresponding to a fractal molecular structure, with the vertex set V(G) representing atoms and the edge set E(G) representing carbon–carbon bonds. The degree of a vertex p , denoted by $d_{p}$ , corresponds to the number of bonds incident to the associated atom (Renai and Roy, 2023). For any considered edge e=pq∈E(G) , we have

1. $w^{*}\left(e\right)=d_{p}*d_{q}$

2. $w^{+}\left(e\right)=d_{p}+d_{q}$

### 2.5 Entropy

According to thermodynamics the term entropy represent a disorder of a system considered. More precisely, depending on the rise in temperature of a matter leads to the deformation of its present state to another state. The highest entropy value we obtained the more energy loss could be predicted. Claude Shannon created Shannon’s information entropy in 1948 to quantify the uncertainty or unpredictability in a piece of data. It measures the typical amount of data produced by a data source. Shannon’s entropy determines the expected information value for each possible outcome for a random variable based on its probability.

The entropy formula produces a value that is often expressed in bits by adding the products of the probability of each result and its logarithm. Since it establishes the bounds of data compression and the effectiveness of communication networks, this idea is crucial to information theory. Shannon’s information entropy is important in information theory and other areas because of a number of important features. Firstly, since entropy is always positive, there can never be less than zero uncertainty or information content. Second, the highest amount of uncertainty is shown by a uniform distribution, where all outcomes have an equal probability of occurring. This is where entropy reaches its maximum. Thirdly, since entropy is cumulative for independent events, the combined entropy of independent events is equal to the sum of the entropies of each individual event. Because of these characteristics, Shannon’s entropy is an important metric in many fields. Its significance arises from its capacity to establish boundaries for data compressibility and communication system efficiency, acting as a standard for lossless data compression. Entropy is also useful for assessing the information content and unpredictability of cryptographic systems, which helps to guarantee data security. Shannon’s entropy is crucial for data transmission, storage, and many other applications that call for effective information processing and management because it offer a mathematical foundation for comprehending information and uncertainty. Shannon’s entropy is an important tool in data transmission, cryptography, chemical graph theory, and other domains where an understanding of information amount is essential. It does this by evaluating the unpredictability or information content in a dataset. In this study, Shannon’s probabilistic function is employed to calculate entropy measures derived from topological indices across different values of n for the two considered fractal molecular systems.

The descriptors summarized in Table 1 find wide-ranging uses in thermodynamics, chemistry, QSPR/QSAR analyses, as well as related scientific domains. For instance, the atom–bond connectivity index has been shown to exhibit strong correlations (Gutman and Tosovic, 2013). To further capture the structural complexity of molecular graphs, the concept of graph entropy was introduced (Mowshowitz and Dehmer, 2012). Although originally formulated to address challenges in communication and information transmission, graph entropy has since found significant applications in diverse fields, including engineering systems, biological networks, and physical dissipative structures (Sabirov and Shepelevich, 2021). Graph entropies are broadly categorized into two classes: probabilistic and deterministic. Intrinsic measures partition the graph into substructures with identical topological characteristics and then define a probability distribution over these components. Extrinsic measures, by contrast, assign probability distributions directly to graph elements, such as vertices or edges. By applying an entropy function to these distributions, one obtains quantitative values that reflect graph complexity in probabilistic terms (Mowshowitz and Dehmer, 2012).

**TABLE 1:** Vertex connectivity-based topological descriptors expressions.

Indices	Formula
First Zagreb	$M_{1}\left(G\right)=\underset{pq\in E\left(G\right)}{\sum }\left[w^{+}\left(e\right)\right]$
Second Zagreb	$M_{2}\left(G\right)=\underset{pq\in E\left(G\right)}{\sum }\left[w^{*}\left(e\right)\right]$
General Randić	$R_{\alpha }\left(G\right)=\underset{pq\in E\left(G\right)}{\sum }{\left[w^{*}\left(e\right)\right]}^{\alpha }$
Randić	$R\left(G\right)=\underset{pq\in E\left(G\right)}{\sum }{\left[w^{*}\left(e\right)\right]}^{-\frac{1}{2}}$
Hyper Zagreb	$HM\left(G\right)=\underset{pq\in E\left(G\right)}{\sum }{\left[w^{+}\left(e\right)\right]}^{2}$
Sum Connectivity	$SCI_{1}\left(G\right)=\underset{pq\in E\left(G\right)}{\sum }\frac{1}{\sqrt{w^{+}\left(e\right)}}$
General Sum Connectivity	$\chi_{\alpha }\left(G\right)=\underset{pq\in E\left(G\right)}{\sum }{\left[w^{+}\left(e\right)\right]}^{\alpha }$
Geometric Arithmetic	$GA\left(G\right)=\underset{pq\in E\left(G\right)}{\sum }\frac{2\sqrt{w^{*}\left(e\right)}}{w^{+}\left(e\right)}$
Atom Bond Connectivity	$ABC\left(G\right)=\underset{pq\in E\left(G\right)}{\sum }\sqrt{\frac{w^{+}\left(e\right)-2}{w^{*}\left(e\right)}}$
Harmonic	$H\left(G\right)=\underset{pq\in E\left(G\right)}{\sum }\frac{2}{w^{+}\left(e\right)}$

Among the various formulations of probabilistic entropy, Shannon’s method remains the most widely applied. In this approach, probability operators are assigned to each information unit, denoted as $k_{1},k_{2},\ldots ,k_{n}$ . The corresponding Shannon entropy, h, serves as the fundamental measure of informational uncertainty and is formally defined as: $SE=-\sum_{i=1}^{n}p\left(k_{i}\right)\log\left(p\left(k_{i}\right)\right)$ where $p\left(k_{i}\right)=\frac{M_{i}}{M}$ , M is the total length of the information, and $M_{i}$ is the total count of the symbol $k_{i}$ in the information (Sabirov and Shepelevich, 2021). This measure is adjusted when applied to chemical graphs to describe their topology. The entropy, measured using the topological index D , is defined as follows (Kazemi, 2016; Mowshowitz and Dehmer, 2012; Sabirov and Shepelevich, 2021).

When adapted to chemical graphs, this formulation is modified to characterize molecular topology. Specifically, the edges of the chemical graph are regarded as the fundamental elements, and probability values are assigned to them through appropriate topological descriptors. Accordingly, the entropy associated with a given topological index D, is expressed as follows (Kazemi, 2016; Mowshowitz and Dehmer, 2012; Sabirov and Shepelevich, 2021).

$$ENT_{D}\left(G\right)=\log\left(D\right)-\frac{1}{D}\underset{ab\in E\left(G\right)}{\sum }j\left(e\right)\log j\left(e\right)$$ (1)

All calculations of topological indices and entropy values were performed using custom-developed codes in MATLAB software. The molecular graphs of Kekulene and terpyridine-based Sierpinski triangle systems were represented through their adjacency matrices, and degree distributions were derived accordingly. Edge partitions were systematically obtained and used as inputs for computing degree-based topological indices such as Zagreb, Randic, Hyper-Zagreb, and ABC indices. Shannon entropy values were then calculated numerically by applying the defined probability functions to these descriptors.

## 3 Results

### 3.1 Computation of degree based topological indices of fractal molecular structures

**FIGURE 2:**
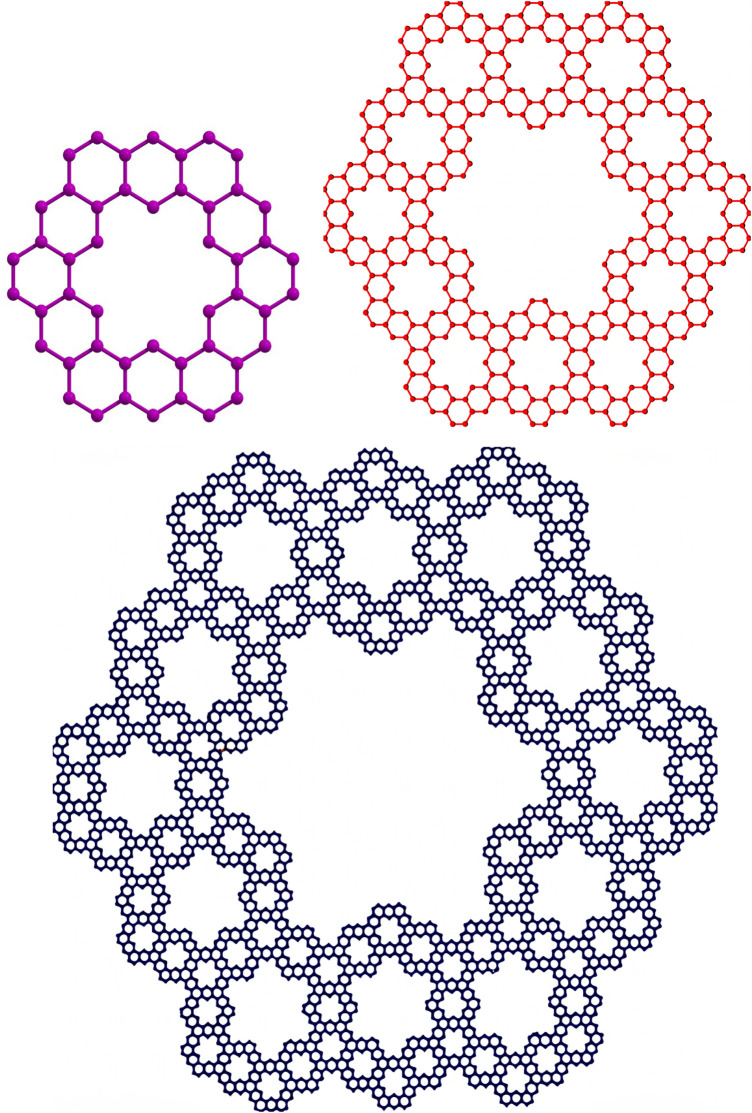
Kekule fractal topology with dimensions n=1,2,3 .

**FIGURE 3:**
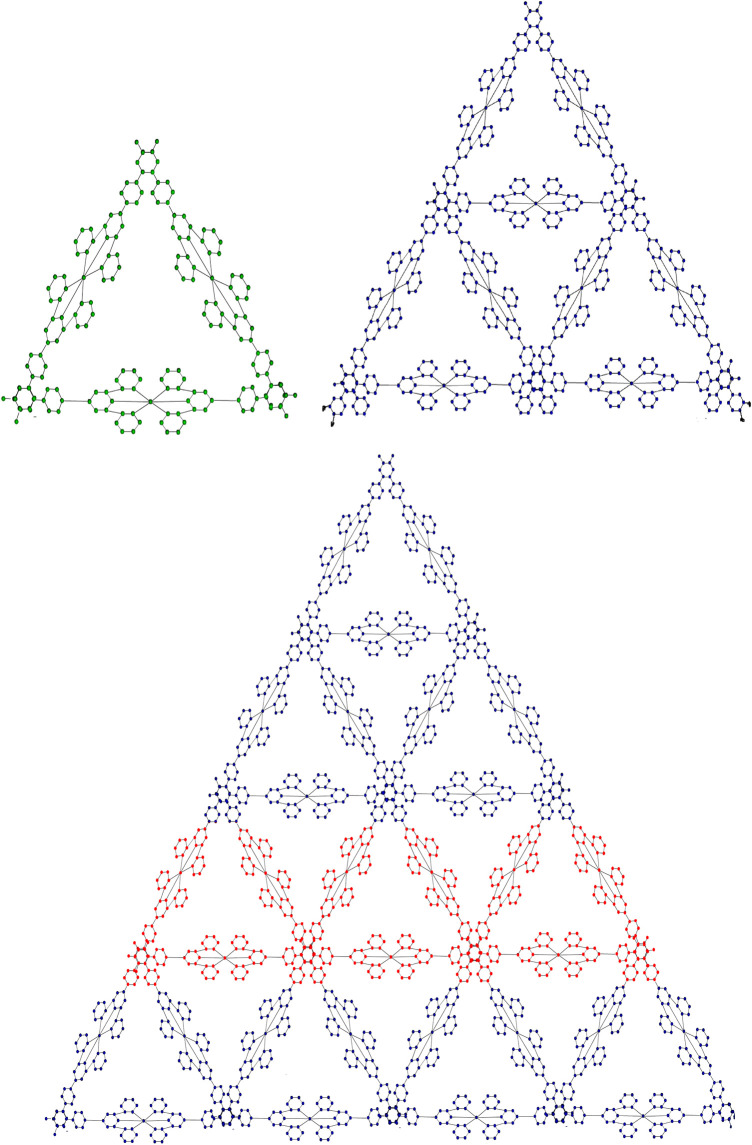
Terpyridine complex Sierpinski triangle fractal topology with dimensions n=1,2,3 .

- Based on the expressions and edge partitions provided in Tables 1–3, the topological indices of the fractal molecular compounds are computed and tabulated. Then the corresponding entropy measures are further computed and illustrated through diagrams to analyze and compare the trends associated with different topological descriptors.
- Graphical representations of the two fractal molecular compounds under consideration are depicted in Figures 2, 3.
- The edge partitions of $KE_{n}$ and $SE_{n}$ molecular graphs are given in Tables 2, 3 respectively.

**TABLE 2:** Edge partitions of kekulene graph $KE_{n}$ .

Edge type	$\left|E_{i}\left(KE_{n}\right)\right|$
(2,2)	$|E_{1}|=75n^{2}-207n+138$
(2,3)	$|E_{2}|=\frac{9}{2}8^{n}$
(3,3)	$|E_{3}|=\frac{3}{2}12^{n}$

**TABLE 3:** Edge partitions of terpyridine Sierpinski triangle graph $SE_{n}$ .

Edge type	$\left|E_{i}\left(SE_{n}\right)\right|$
(1,3)	$|E_{1}|=4n+2$
(2,2)	$|E_{2}|=24n^{2}+25n-1$
(2,3)	$|E_{3}|=36n^{2}+38n+10$
(3,3)	$|E_{4}|=27n^{2}+30n-3$
(3,6)	$|E_{5}|=9n^{2}+9n$

Theorem 3.1 Let $KE_{n}$ be the n dimensional Kekulene fractal graph. Then

$$\begin{array}{ll} & 1.\;M_{1}=300n^{2}-828n+\frac{45\cdot 8^{n}}{2}+9\cdot 12^{n}+552\\ & 2.\;M_{2}=300n^{2}-828n+27\cdot 8^{n}+\frac{27\cdot 12^{n}}{2}+552\\ & 3.\;R_{\alpha }=4^{\alpha }\left(75n^{2}-207n+138\right)+\frac{9\cdot 6^{\alpha }\cdot 8^{n}}{2}+\frac{3\cdot 9^{\alpha }\cdot 12^{n}}{2}\\ & 4.\;R=\frac{3\cdot \sqrt{6}\cdot 8^{n}}{4}-\frac{207n}{2}+\frac{75n^{2}}{2}+\frac{12^{n}}{2}+69\\ & 5.\;\chi_{\alpha }=4^{\alpha }\left(75n^{2}-207n+138\right)+\frac{9\cdot 5^{\alpha }\cdot 8^{n}}{2}+\frac{3\cdot 6^{\alpha }\cdot 12^{n}}{2}\\ & 6.\;SCI=\frac{9\cdot \sqrt{5}\cdot 8^{n}}{10}-\frac{207n}{2}+\frac{6^{\frac{1}{2}}\cdot 12^{n}}{4}+\frac{75n^{2}}{2}+69\\ & 7.\;HM=1200n^{2}-3312n+\frac{225}{2}\cdot 8^{n}+54\cdot 12^{n}+2208\\ & 8.\;GA=\frac{9\cdot \sqrt{6}\cdot 8^{n}}{5}-207n+75n^{2}+\frac{3\cdot 12^{n}}{2}+138\\ & 9.\;ABC=\frac{\sqrt{2}\;\left(75n^{2}-207n+138\right)}{2}+\frac{9\sqrt{2}\;8^{n}}{4}+12^{n}\\ & 10.\;H=\frac{75n^{2}}{2}-\frac{207n}{2}+\frac{9\cdot 8^{n}}{5}+\frac{12^{n}}{2}+69 \end{array}$$

Proof. The edges of molecular graph are partitioned according to the degrees of their terminal vertices. Using the edge partitions listed in Table 2 together with the index formulations provided in Table 1, the corresponding topological indices are computed.

Theorem 3.2 Let $SE_{n}$ be the n dimensional terpyridine complex Sierpinski triangle graph. Then

$$\begin{array}{ll} & 1.\;M_{1}=519n^{2}+567n+36,\\ & 2.\;M_{2}=717n^{2}+772n+35,\\ & 3.\;R=\frac{45n}{2}+\frac{\sqrt{18}\left(9n^{2}+9n\right)}{18}+\frac{\sqrt{6}\left(36n^{2}+38n+10\right)}{6}+21n^{2}+\frac{\sqrt{3}\left(4n+2\right)}{3}-\frac{3}{2},\\ & 4.\;R_{\alpha }=3^{\alpha }\left(4n+2\right)+18^{\alpha }\left(9n^{2}+9n\right)+4^{\alpha }\left(24n^{2}+25n-1\right)+9^{\alpha }\left(27n^{2}+30n-3\right)+6^{\alpha }\left(36n^{2}+38n+10\right),\\ & 5.\;SCI_{1}=\frac{35n}{2}+\frac{\sqrt{6}\left(27n^{2}+30n-3\right)}{6}+\frac{\sqrt{5}\left(36n^{2}+38n+10\right)}{5}+15n^{2}+\frac{1}{2},\\ & 6.\;\chi_{\alpha }=4^{\alpha }\left(4n+2\right)+9^{\alpha }\left(9n^{2}+9n\right)+4^{\alpha }\left(24n^{2}+25n-1\right)+6^{\alpha }\left(27n^{2}+30n-3\right)+5^{\alpha }\left(36n^{2}+38n+10\right),\\ & 7.\;HM=2985n^{2}+3223n+158,\\ & 8.\;GA=55n+\frac{2\sqrt{2}\left(9n^{2}+9n\right)}{3}+\frac{2\sqrt{6}\left(36n^{2}+38n+10\right)}{5}+51n^{2}+\frac{\sqrt{3}\left(4n+2\right)}{2}-4,\\ & 9.\;ABC=\frac{\sqrt{7}\left(9n^{2}+9n\right)}{\sqrt{18}}+\frac{60n^{2}+63n+9}{\sqrt{2}}+\frac{\sqrt{2}\left(4n+2\right)}{\sqrt{3}}+18n^{2}+20n-2\\ & 10.\;H=\frac{187n^{2}}{5}+\frac{417n}{10}+\frac{7}{2}. \end{array}$$

Proof. The edges are partitioned according to the degrees of their incident vertices. Utilizing the edge partitions provided in Table 3, together with the index equations summarized in Table 1, the corresponding topological indices are calculated.

## 4 Discussion

### 4.1 Computational strategies for computing topological indices based entropy measures and numerical values

- Based on the computed degree-based topological descriptors, the entropy values are obtained using Shannon’s method by defining an appropriate probability function.
- Shannon’s entropy equation is applied to evaluate these measures, with the procedure illustrated through the calculation of entropy values for the fractal structures using the Hyper Zagreb index.
- Let G denote the fractal Sierpinski triangle system $SE_{n}$ . The computation of entropy values corresponding to the Hyper Zagreb index, using Equation 1, is presented below (Renai and Roy, 2023).

$$ENT_{HM}\left(G\right)=\log\left(HM\left(G\right)\right)-\frac{1}{HM\left(G\right)}\underset{ab\in E\left(G\right)}{\sum }{\left[w^{+}\left(e\right)\right]}^{2}\log{\left[w^{+}\left(e\right)\right]}^{2}$$

Through the substitution of the edge partitions provided in Table 2 into the respective formulations, we obtain the following,

$$\begin{array}{ll} ENT_{HM}\left(G\right) & =\log\left(2985n^{2}+3223n+158\right)-\left(1/\left(2985n^{2}+3223n+158\right)\right)\\ & \left(\left(4n+2\right)\left(16\right)\;\log\left(16\right)+\left(24n^{2}+25n-1\right)\left(16\right)\;\log\left(16\right)+\left(36n^{2}+38n+10\right)\right.\\ & \left.\left(25\right)\;\log\left(25\right)+\left(27n^{2}+30n-3\right)\left(36\right)\;\log\left(36\right)+\left(9n^{2}+9n\right)\left(81\right)\;\log\left(81\right)\right)\\ ENT_{HM}\left(G\right) & =\log\left(2985n^{2}+3223n+158\right)-\left(10645.4n^{2}+11421.7n+462.3\right)/\left(2985n^{2}+3223n+158\right) \end{array}$$

**FIGURE 4:**
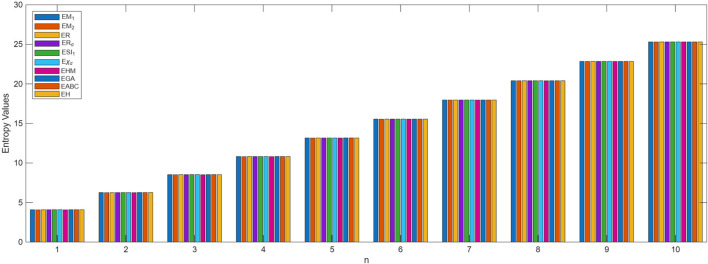
Graphical representation of $KE_{n}$ entropy values.

**FIGURE 5:**
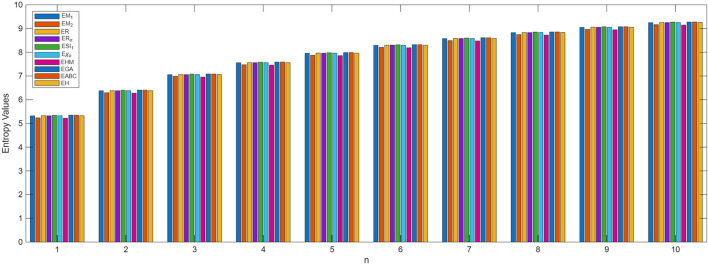
Graphical representation of $SE_{n}$ entropy values.

The process of deriving a comprehensive entropy expression using Shannon’s approach would require a substantial explanation if formulated as theorems. However, individuals can effectively apply the outlined strategy for entropy computation across various topological indices for chemical structures. The subsequent discussion delves into numerical entropy values (refer Tables 4, 5), and graphical representations (see Figures 4, 5) of both fractal compound structures, as detailed.

**TABLE 4:** Degree based numerical entropy values of $KE_{n}$ .

n	$EM_{1}$	$EM_{2}$	ER	$ER_{\alpha }$	ES1	$ES_{\alpha }$	EHM	EGA	EABC	EH
1	4.0876	4.0655	4.0869	4.0869	4.0926	4.0926	4.0683	4.0943	4.0939	4.0872
2	6.2634	6.2437	6.2624	6.2624	6.2676	6.2676	6.2472	6.2690	6.2686	6.2631
3	8.5291	8.5102	8.5277	8.5277	8.5331	8.5331	8.5136	8.5346	8.5342	8.5285
4	10.8165	10.8017	10.8152	10.8152	10.8196	10.8196	10.8048	10.8206	10.8202	10.8161
5	13.1615	13.1499	13.1603	13.1603	13.1639	13.1639	13.1525	13.1648	13.1644	13.1611
6	15.5464	15.5375	15.5453	15.5453	15.5483	15.5483	15.5394	15.5489	15.5486	15.5460
7	17.9597	17.9530	17.9587	17.9587	17.9611	17.9611	17.9545	17.9616	17.9613	17.9593
8	20.3940	20.3892	20.3933	20.3933	20.3950	20.3950	20.3902	20.3954	20.3952	20.3937
9	22.8438	22.8404	22.8432	22.8432	22.8445	22.8445	22.8411	22.8448	22.8446	22.8435
10	25.3045	25.3022	25.3042	25.3042	25.3050	25.3050	25.3027	25.3052	25.3051	25.3044

**TABLE 5:** Degree based numerical entropy values of $SE_{n}$ .

n	$EM_{1}$	$EM_{2}$	ER	$ER_{\alpha }$	EHM	$ESCI_{1}$	$E\chi_{\alpha }$	EGA	EABC	EH
1	5.3189	5.2337	5.3256	5.3210	5.2198	5.3413	5.3255	5.3467	5.3459	5.3256
2	6.3748	6.2903	6.3821	6.3774	6.2746	6.3976	6.3813	6.4033	6.4025	6.3813
3	7.0527	6.9868	7.0603	7.0555	6.9522	7.0757	7.0592	7.0815	7.0807	7.0593
4	7.5557	7.4718	7.5634	7.5586	7.4550	7.5787	7.5622	7.5846	7.5838	7.5623
5	7.9564	7.8726	7.9642	7.9594	7.8556	7.9794	7.9629	7.9853	7.9845	7.9629
6	8.2896	8.2059	8.2975	8.2966	8.1888	8.3127	8.2961	8.3185	8.3178	8.2962
7	8.5749	8.4913	8.5829	8.5780	8.4741	8.5980	8.5815	8.6093	8.6087	8.5815
8	8.8245	8.7409	8.8324	8.8276	8.7236	8.8476	8.8310	8.8535	8.8527	8.8311
9	9.0463	8.9627	9.0542	9.0494	8.9453	9.0694	9.0528	9.0753	9.0745	9.0528
10	9.2458	9.1623	9.2538	9.2490	9.1449	9.2689	9.2523	9.2748	9.2741	9.2524

This study demonstrates that entropy, based on degree-based topological descriptors, acts as a sensitive measure of structural complexity in fractal molecular systems. This method, compared to traditional entropy estimates reliant on electron density distributions, just rely on the fundamental graph structure, making it computationally efficient and broadly accessible. The consistent increase in entropy observed in successive generations of fractal designs emphasizes the hierarchical evolution and enhanced connectedness inherent to these systems. These developments are particularly relevant for QSPR and QSAR research, where entropy functions as an interpretable descriptor connecting molecule structure to physical properties.

## 5 Conclusion

This study investigated the utilization of Shannon entropy in fractal chemical structures by the application of vertex degree-based topological descriptors. The proposed framework combines graph-theoretical invariants with information-theoretic measures, providing a new approach to defining the structural complexity of self-similar molecular architectures. The entropy patterns seen over many fractal generations indicate that structural development is associated with significant alterations in information content, thereby establishing a quantifiable connection between topology and complexity. This entropy-based approach is not merely of theoretical significance; it provides a basis for predictive modeling in QSPR/QSAR research. This technique may be beneficial in supramolecular chemistry, and the creation of sophisticated functional materials that often exhibit fractal-like structures. The current study focuses on degree-based descriptors; however, the methodology can be extended to include additional structural parameters and different entropy measures, thereby facilitating future research on the relationship between molecular topology, information theory, and physicochemical properties.

## Data Availability

The original contributions presented in the study are included in the article/supplementary material, further inquiries can be directed to the corresponding author.
